# Transtinib, a potent tyrosine kinase inhibitor inhibits L858R/T790M mutant NSCLC cell lines and xenografts

**DOI:** 10.18632/oncotarget.7140

**Published:** 2016-02-02

**Authors:** Peng Hu, Da-xiong Han, Run-sheng Ruan, Li-Mou Zheng, Shiu-Huey Chou, Chi-Meng Tzeng

**Affiliations:** ^1^ Translational Medicine Research Center, School of Pharmaceutical Science, Xiamen University, Xiamen, P.R. China; ^2^ Key Laboratory for Cancer T-Cell Theranostics and Clinical Translation (CTCTCT), Xiamen P.R. China; ^3^ Department of Life Science, Fu-Jen Catholic University, Xinzhuang District, New Taipei City, Taiwan

**Keywords:** epidermal growth factor receptor (EGFR), tyrosine kinase inhibitors (TKIs), anilinoquinazoline, irreversible, transtinib

## Abstract

Non-small cell lung cancer (NSCLC) patients with activating epidermal growth factor receptor (EGFR) mutations initially respond well to the EGFR tyrosine kinase inhibitors (TKIs) erlotinib and gefitinib. However, clinical efficacy is limited by the development of resistance. In most cases, this resistance is in the form of the T790M mutation. Here, we report the design, synthesis and biochemical evaluation of a novel series of irreversible EGFR tyrosine kinase inhibitors (EGFR-TKIs) that are derived from the anilinoquinazoline scaffold. Guided by molecular modeling, this series of analogs was evolved to target a cysteine residue in the ATP binding site via covalent bond formation and to achieve high levels of anti-tumor activity in cell cultures and in xenografts. The most promising compound 13c ((E) –N - (4 - (4 - (3-fluorobenzyloxy) -3- chlorophenylamino) -7-ethoxyquinazolin-6-yl) -3- ((S) -pyrrolidin-2-yl)acrylamide, which we named Transtinib) displayed strong anti-proliferative activity against the H1975 and A431 cell lines with IC_50_ values of 34 nM and 62 nM, respectively. In xenograft models, Transtinib significantly decreases tumor size for a prolonged period of time. These results suggest that Transtinib is a potential cancer therapeutic drug lead for the inhibition of mutant EGFR to overcome the development of resistance.

## INTRODUCTION

The role of the epidermal growth factor receptor (EGFR) in non-small cell lung cancers (NSCLC) is well-known. Inhibition of the kinase domain of EGFR and the resultant oncogenic cell signaling disruption by small molecule inhibitors have been shown to be particularly beneficial in patients carrying the so-called “sensitizing mutations” such as L858R and the exon-19 deletion [1, 2], which contribute to nearly 90% of lung-cancer-specific EGFR mutations [3, 4]. Unfortunately, approximately 70% of patients respond initially but develop resistance with a median time to progression of 10-16 months [2, 5–8]. In at least 50% of these resistance cases, the emergence of a secondary gate keeper mutation, T790M in exon 20 of EGFR, occurs in combination with an activating mutation [9].

Over the past several decades, various approaches have been developed to target the EGFR signaling pathway, including monoclonal antibodies and small molecule tyrosine kinase inhibitors (TKIs). Small molecule inhibitors that target EGFR are undoubtedly successful, and the first-generation EGFR tyrosine kinase inhibitors (TKIs) (Table 1) such as gefitinib (IRRESA) [10], elotinib (TARCEVA) [11], lapatinib (TYKERB) [12], and icotinib (CONMANA) [13] have efficacy against several types of human cancers. Gefitinib, erlotinib and icotinib were approved for the treatment of NSCLC, while lapatinib, which inhibits EGFR and HER2 kinases, is used in combination therapy for HER2-positive metastatic breast cancer.

**Table 1:** Transtinib and other TKIs

The chemical name or trade name	The structure of the chemical	The drug targets and its IC_50_(nM)	Interaction with target	Status	Company	Reference
IRESSA (Gefitinib, ZD1839)	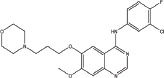	Mutant EGFR(HER1) IC_50_=33 nM	Reversible	Approved by FDA in 2003 for the treatment of NSCLC	AstraZeneca	[10]
TARCEVA (Erlotinib, OSI-774)	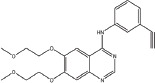	Mutant EGFR(HER1) IC_50_=2 nM	Reversible	First approved by FDA in 2004 for pancreatic cancer and then in 2013 for the first-line treatment of metastatic NSCLC with activating mutant EGFR	Roche	[11]
TYKERB (lapatinib, GW2016)	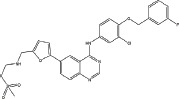	A dual inhibitor mutant EGFR, IC_50_=10.2 nM HER2, IC_50_=9.8 nM	Reversible	First approved by FDA in 2007 for breast cancer and in 2012 in combination with trastuzumab for the treatment of HER2-positive metastatic breast cancer in patients that have received prior trastuzumab therapy	GlaxoSmithKline plc	[12]
CONMANA (Icotinib)	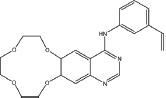	Mutant EGFR IC_50_=5 nM	Reversible	Approved by SFDA in 2011 for NSCLC	BETA PHARMA (China)	[13]
XL647(KD019)	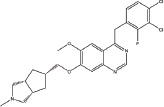	EGFR, IC_50_= 0.3 nM ErbB2, IC_50_=16 nM KDR, IC_50_=1.5 nM EphB4, IC_50_= 1.4 nM	Reversible	Study of the combination of KD019 and Trastuzumab in subjects with HER2 positive metastatic breast cancer	Kadmon Corporation LLC	[28]
HKI-272	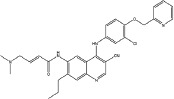	EGFR, IC_50_=90 nM HER2, IC_50_=60 nM	Irreversible	A phase 1/2 clinical trial in combination with Trastuzumab or Taxol for advanced breast cancer	Pfizer	[27]
GILOTRIF (Afatinib, BIBW2992)	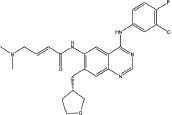	EGFR(L858R/T790M), IC_50_=10 nM HER2, IC_50_=14 nM	Irreversible	Approved by FDA in 2013 for first-line treatment of metastatic NSCLC with mutant EGFR	Boehringer Ingelheim	[17]
AZD9291	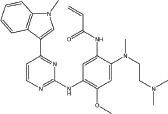	EGFR(L858R), IC50=12 nM EGFR(L858R, T790M), IC50=1 nM	Irreversible	Clinical trials study in NSCLC	AstraZeneca	[29]
Transtinib	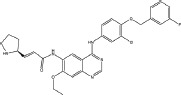	--	Irreversible	This study	This study	This study

To address the resistance of the first -generation of reversible TKIs, a number of second-generation irreversible inhibitors were developed based on the anilinoquinazoline template of gefitinib (Table 1). These inhibitors contained an acrylamide substituent, a Michael acceptor system that takes advantage of the covalent modification of Cys-797 located at the lip of the ATP binding clef of EGFR, which is thought to overcome the resistance of the first-generation inhibitors. These agents were shown, in pre-clinical models, to be more potent against the second-site mutation than gefitinib or erlotinib [14]. The following are several second generation inhibitors: [(E)-N-(4-(4- ((pyridine – 2 – yl) methoxy) – 3 -chlorophenylamino) -3-cyano-7- ethoxyquinolin-6-yl) -4-(dimethylamino)but-2-enamide (HKI-272) [15], (E) – N - (7 - ((R) - tetrahydrofuran-3-yloxy) -4-(3-chloro-4- fluorophenylamino) quinazolin-6-yl) – 4 - (dimethylamino)but-2-enamide (BIBW2992) [16, 17], and (E)-N-(4- (3-chloro-4- fluorophenylamino) - 7-methoxyquinazolin-6-yl) -4-(piperidin-1-yl)but-2-enamide (PF-00299804) [18, 19]. However, most of these irreversible EGFR inhibitors failed in clinical-trials because of their toxicities. Thus, clinically acquired drug resistance against EGFR kinase inhibitors is a major challenge in targeted cancer therapies for NSCLC treatment. Herein, we describe some of our work that led to the identification of the clinical candidate Transtinib(13c), a potent inhibitor of both sensitizing and double mutant forms of EGFR.

We focused our development on modifications to the solvent region by attaching cyclic systems to the anilinoquinazoline ring. Therefore, a series of novel EGFR inhibitors were designed and synthesized by incorporating acrylamide-based side chains into the C6 position and alkoxy-based side chains into the C7 position of the 4-anilinoquinazoline scaffold. Different types of anilines from gefitinib, erlotinib, lapatinib and other advanced EGFR inhibitors were substituted into the C-4 position of the quinazoline template. As Edgar R. Wood et.al [20] described, the pyrrolidine is a structural feature that is particularly adept at assisting in protein alkylation in the active site. In our candidate compounds, we predicted that pyrrolidine would promote the formation of covalent binding. Therefore, we introduced a pyrrolidine substituent into the covalent binding structures.

## RESULTS AND DISCUSSION

### Chemical names and structures

**Scheme 1:**
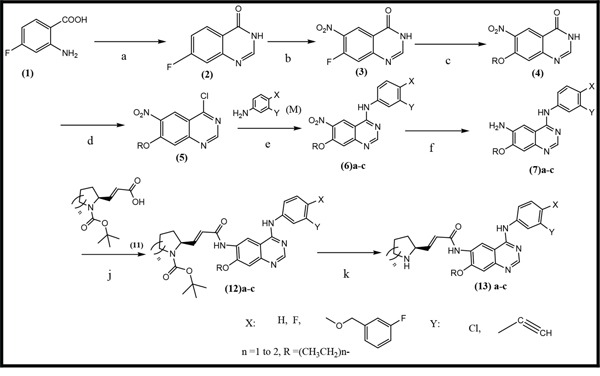
General synthetic approach to the designed compounds Reagents, conditions and yields of each procedure are as follows: (a) HCONH2 130-140°C, 20 h, 80%; (b) HNO3 /H2SO4, 0-20-110°C, 91%; (c) Na, CH3CH2OH, reflux, 2.5 h, 73%; (d) SOCl2, POCl3, DMF, 80°C, 3.5 h, 97%; (e) isopropanol, reflux 2 h, 80-90%; (f) Fe, CH3CH2OH, CH3COOH, reflux, 4 h, 75-80%; (j) C2Cl2O2, CHCl3, DMF, NEt3, reflux, 3 h; (k) CF3COOH, 20°C, 1 h. All heating processes were carried out in an oil bath, and the total yield of 13c (Transtinib) was 60%.

**Scheme 2:**
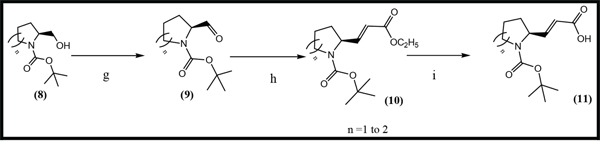
General synthetic approach to the R3 substituent group Reagents and conditions are as follows: (g) CH2Cl2, C2Cl2O2, DMSO, NEt3, −78°C, 2 h, [21] (h) THF, NaH, C8H17O5P, N2, 0°C, 2h,[22] (i) CH3CH2OH, LiOH, 15 h[23].

**Figure 1:**
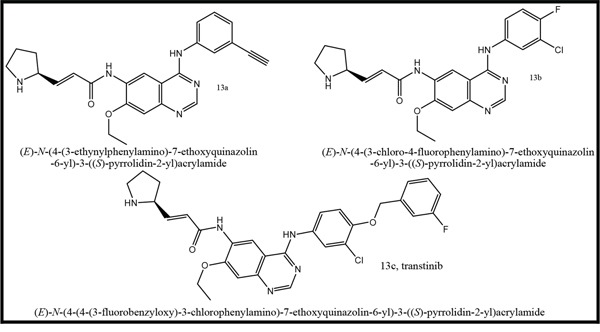
Structures and names of the synthesized compounds (13a-c)

Three anilinoquinazoline compounds (13a-c) were synthesized based on Scheme 1 and 2. To access compound 11, commercial (S)-(-)-1-Boc-2-pyrrolidinemethanol with n=1 was used. To access compounds13a-c, R was setted with n=1. All the structures and names are as follows (Figure 1).

### Transtinib potently and selectively targets mutant EGFR cell lines in vitro

Transtinib and other two compounds (13a and, 13b) inhibit cell proliferation in cell lines expressing wild type and mutant EGFR. The in vitro potency of the compounds in comparison to gefitinib was determined in three cancer cell lines. Cell proliferation assays were performed using the MTT assay with increasing concentrations of compounds for 72 h. The results expressed as IC_50_ are summarized in Table 2. As shown in Table 2, three synthesized compounds demonstrated moderate to excellent anti-proliferative activities against different cancer cell lines, with IC_50_ values ranging from 34 nM to 95 nM (Table 2). All three compounds were more potent than gefitinib at activating mutation and the gatekeeper mutation (T790M). As expected, none of the compounds, including gefitinib, showed good efficacy against the SW620. In particular, consistent with molecular modeling in the ATP pocket of EGFR, compound 13c (Transtinib) was found to possess higher antitumor activities with an IC_50_ of 34 nM in the H1975 cell line with both the sensitive mutation L858R and the resistant mutation T790M. Moreover, transtinib exhibited moderate potency against the WT EGFR cell line (A431) with an IC_50_ of 62 nM.

**Table 2:** Anti-cancer properties of Transtinib and its derivates in cell models. The IC_50_ values are the averages of at least three independent experiments

Cell lines	Mutation	gefitinib IC_50_(nM)	13a IC_50_(nM)	13b IC_50_(nM)	13c(transtinib) IC_50_(nM)
**H1975**	L858R/T790M	>1000	43±12.8	50±8.2	34±7.6
**A431**	Wild type(over-express)	85±8.8	95±13.4	75±9.8	62±8.3
**N87**	HER2(over-express)	>1000	87±8.2	80±10.2	47±8.8
**SW620**	No HER2 and EGFR express	>1000	>1000	>1000	>1000

In addition to assessing the activity against the activating and resistant mutants, we similarly assessed the potency of compounds against the HER2 over-expressing cell line (N87). N87 cells were treated with increasing concentrations of either gefitinib, or 13a, 13b, or transtinib for 72 h. All compounds were effective against WT HER2, except for gefitinib. Transtinib exhibited was more effective at inhibiting the growth of this cell line than 13a or 13b with an IC_50_ of 47 nM. Thus, transtinib may also be able to target HER2 in tumors. These results indicated that the acrylamide and pyrrolidine substituent groups may increase the binding affinity between compounds and EGFR. Therefore, transtinib was selected for further docking and xenograft model studies.

### Transtinib likely docks into EGFR in a similar manner as HKI272

To give structural insight into the ligand/enzyme interactions and an explanation of the observed activity, we examined the interaction of ligands with the EGFR complex structure by molecular docking of compounds into the ATP binding site of EGFR.

**Figure 2:**
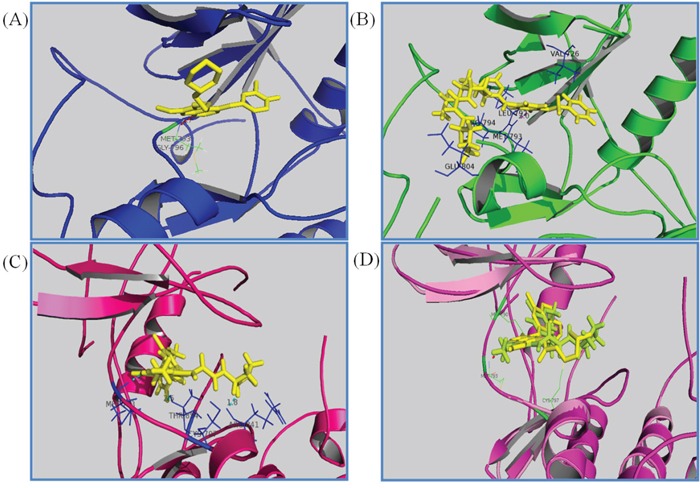
Drug docking studies in the WT and mutant EGFR kinases The binding mode of Transtinib (B, C, D), gefitinib (A) and HKI272 (D) are compared in the WT (A, B) and T790M mutation kinases (C, D). Key interactions are labeled, the inhibitors are shown in stick form, and hydrogen bonds are indicated with dashed lines. **A.** gefitinib binding with the active form of EGFR (2ITY [10]), **B.** Transtinib binding with the active form of EGFR, hydrogen bond formed between the Cl substituent and the Met793 residue **C.** Transtinib binding with the C-helix out inactive mode with the T790M mutation of EGFR, three hydrogen bonds were formed with Met793, Arg841 and Thr854. **D.** HKI272 (yellow) superposed on Transtinib (lemon) in 2JIV.

When docking with the WT active state of EGFR, Transtinib (Figure 2B) was more potent than gefitinib (Figure 2A). The quinazoline ring is oriented with the 1-N back of the ATP binding pocket, a hydrogen bond formed between the Cl substituent and the Met793 residue, and the van der Waals forces occur among the side chain of Transtinib and the residues Val726, Leu792, Glu804, and Pro794. While docking with the DFG-out state, bearing the T790M mutation, all of the docking results were poor (data not shown). Thus, we adopted the C-helix out inactive mode, and as illustrated in Figure 2C and 2D, our analysis suggested that Transtinib favorably fitted into EGFR in a similar manner as HKI272. Moreover, a number of key interactions with the mutant EGFR (T790M) protein likely contributed to this compound's potency. The quinazoline ring is oriented with the 1-N in the back of the ATP-binding pocket, and the covalent Michael acceptor (acrylamide group) is located in close proximity to Cys797, which is feasible for covalent bond formation. Furthermore, the quinazoline core of the inhibitor forms hydrogen bond (1.9 Å) interactions with the backbone amide of the hinge residue Met790 directed toward the gatekeeper. Two other hydrogen bonds form among the side chain of Transtinib and the residues Arg841 (1.8 Å) and Thr854 (2.5 Å) of EGFR. We speculated that the pyrrolidine group may have potential advantages for the interaction with Arg841 and Thr854, which would further strengthen the ligand's affinity towards the gatekeeper mutant.

### Transtinib demonstrates a significant prolonged response in mutant EGFR xenograft models *in vivo*

**Figure 3:**
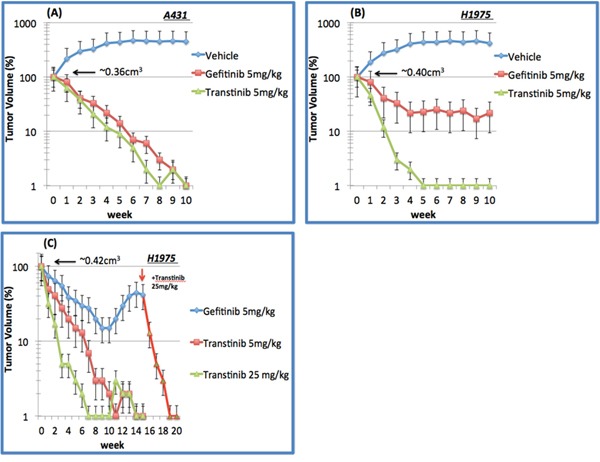
*In vivo* tumor suppression of Transtinib in xenograft models of EGFR-TKI sensitizing A431 and T790M/L858R resistant H1975 non-small cell lung cancer **A.** A431 and **B.** H1975 xenograft following 10 weeks of daily 5 mg/kg gefitinib (n=6) and Transtinib treatment (n=8 and 10 mice, respectively). **C.** H1975 following chronic daily oral dosing of 5 and 25 mg/kg Transtinib (n=10 and 8, respectively). Additionally, 25 mg/kg Transtinib was applied to the 5 mg/kg Gefitinib treatment group after 15 weeks to restore the anti-cancer efficacy. Data are plotted as the mean ± standard error.

To explore the in vivo activity of Transtinib, we dispensed the drug lead as mono-therapy against mutant EGFR xenografts on behalf of clinical NSCLC scenarios. Once daily dosing of Transtinib induced significant dose-dependent decreases in tumor size in both A431 and H1975 tumor xenograft models, with 90% tumor reduction observed at doses of 5.0 mg/kg/day in both models after 4 weeks (Figure 3A and 3B). Similar tumor reductions were observed after applying of 5 mg/kg/day Gefitinib. These studies showed that Transtinib can induce significant decreases in tumor size at low doses against both EGFR drug-sensitized and T790M/L858R- resistant EGFR mutant disease animal models.

We then challenged the durability of tumor reduction through 16-20 week long- term daily oral dosing of Transtinib in 8-10 H1975 xenografts (Figure 3C). As a comparison, gefitinib at 5 mg/kg/day induced less tumor reduction and tumors began to re-grow after approximately 15 weeks, but an increased dose of 25 mg/kg/day Transtinib triggered tumor reductions, suggesting that re-growth was still driven by T790M/L858R-resistant EGFR mutants. In H1975 xenografts, 5 mg/kg/day Transtinib resulted in almost complete responses in 9 of 10 tumors at week 11. No visible tumors were observed after 7 weeks of dosing at 25 mg/kg/day Transtinib. The complete responses were maintained for the duration of the study period with no tumor recurrence during the 20 weeks of treatment. Moreover, no growth was observed for an additional 5 weeks after Transtinib treatment was terminated.

In comparison, the efficacy against wild-type and mutant EGFR xenografts was examined. Transtinib did moderately inhibit tumor growth in A431. However, this same 5 mg/kg/day dose induced complete tumor reduction in H1975 mutant EGFR tumor xenografts, suggesting that Transtinib possesses a novel selectivity margin over WT EGFR.

## MATERIALS AND METHODS

### Chemistry

A general approach to synthesize the designed quinazoline compounds is shown in Scheme 1, starting from commercially available 2-amino-4-fluorobenzoic acid **(1)**. Unless otherwise noted, all reagents and solvents were purchased from Sigma or Aldrich and used without further purification. Dry solvents were purchased as anhydrous reagents from commercial suppliers.

All of the structures of the compounds were evaluated by ^1^H NMR spectroscopy at 400 MHz or 300 MHz, and by MS (BRUKER Autoflex TOF/TOF). ^1^H chemical shifts are reported in δ (ppm) as s (singlet), d (doublet), dd (doublet of doublet), t (triplet), q (quartet), m (multiplet), and br s (broad singlet) and are referenced to the residual solvent signal: CDCl_3_ (7.26) or DMSO-*d_6_* (2.50). The compounds (11) were synthesized according to Scheme 2.

### Molecular docking study

**Figure 4:**
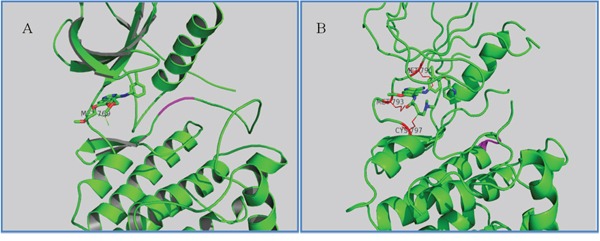
Two inactive states of EGFR **A.** Crystallographic structure of EGFR with erlotinib (1M17, DFG-out state). The interaction residue Met769 is labeled and the DFG motif is labeled in magenta. **B.** Crystallographic structure of EGFR with HKI272 (2JIV, C-helix out inactive mode). The interaction residues Met790/793 and Cys797 are labeled.

The wild type (WT) and various mutant forms of the EGFR kinase domain have been structurally characterized. Analysis of previously published structures of TKI binding to EGFR revealed two binding modes. The first mode is the DFG-out state, which is characterized by the core structure of inhibitors forming strong interactions with the hinge region in EGFR and the other moiety of inhibitors extending to (or close to) the solvent exposure area, such as erlotinib (Figure 4A), gefitinib, and BIBW2992. The second mode is the C-helix out inactive mode. In this second mode, the core structure of inhibitors, such as HKI272 (2JIV) [15] (Figure 4B), forms a single H-bond and hydrophobic interactions with the hinge region, including the mutant gatekeeper residue Met790, while the lipophilic moiety of the inhibitors expands to the back pocket of ATP binding and disrupts the salt bridge between the glutamate residue on helix αC and the lysine residue on the N-lobe. In addition to these noncovalent interactions, the covalent bond is formed between Cys797 and the crotonamide Michael-acceptor group on the inhibitor.

To predict the possible binding mode in the ErbB family enzyme active site, Transtinib underwent *in silico* screening, and the autoDock4.2 package [24] was used for molecular docking. In addition, the structures of HKI-272 and gefitinib were considered as controls. The docking was performed using the receptor from the 2JIV (HKI272) and 2ITZ (gefitinib) structures from the RCSC Protein Data Bank. The co-crystal inhibitors were removed from the initial X-ray structures, water molecules were then removed, and polar hydrogens and Gasteiger charges were added using the Autodock Tool. Ligands were optimized for energy and geometry using MM2 force fields. The rotational bonds of all proteins were regarded as being rigid, while the rotational bonds of the ligands were treated as flexible. All figures were produced using the open source PyMOL 1.5 package.

### Anti-proliferative activities of compounds 13a-13c

To assess their potential antitumor activity, compounds 13a, 13b, and 13c (Transtinib) were tested at a range of concentrations and evaluated in four cell lines harboring either wild-type or mutant EGFR. The first generation TKI (gefitinib) was employed as the positive control. For EGFR (T790M/L858R), the human NSCLC NCI-H1975 was obtained and cultured in RPMI-1640 with 10% fetal bovine serum (FBS) and 1% penicillin-streptomycin. For HER2 expression and no expression of EGFR and HER2, the human gastric carcinoma cell line NCI-N87 (HER2 over-expressing) and the human colorectal adenocarcinoma cell line SW620 (no expression of EGFR and HER2) were obtained from the American Type Culture Collection and cultured in RPMI-1640 with 10% FBS and 1% penicillin-streptomycin. For EGFR (wild-type), the human epidermoid A431 carcinoma cell line (EGFR overexpression) was obtained and cultured in high-glucose DMEM with 10% FBS and 1% penicillin-streptomycin. The ability of the compounds to inhibit proliferation was measured by the MTT assay. Cell lines were plated in 96-well plates at between 1,000 and 10,000 cells per well. The cells were allowed to attach overnight at 37°C under 5% CO_2_. Twenty-four hours after the cells were plated, they were exposed to compounds at titrations concentration and the treated cells were incubated for a further 72 h at 37°C under 5% CO_2_. Fresh MTT was added to each well at a terminal concentration of 5 mg/mL and incubated with the cells at 37°C for 4 h. The formazan crystals were dissolved by adding 100μl of DMSO to each well, and the absorbance at 492 nm (for the absorbance of MTT formazan) and 630 nm (for the reference wavelength) were measured with the ELISA reader. All of the compounds were tested three times in each of the cell lines. The results were expressed as IC_50_ (inhibitory concentration 50%) and were the averages of three determinations.

### Transtinib anti-tumor activity in *in vivo* xenograft models

To explore the in vivo activity of transtinib, we administered the drug as monotherapy against various mutant EGFR xenografts representing clinical NSCLC scenarios.

Four-six-week-old SCID female nude mice were purchased from the Animal facility, Institute of Molecular Biology, Academic Sinica, and housed in ventilated cages. All standard methods for animal model and assays were approved by the School of Pharmaceutical Sciences, Xiamen University. Xenografts were bred by injecting 5~7×10^6^ cells (H1975 and A431) with 50% Matrigel in total volume of 0.5 ml per mice. Before treatment, mice were randomized to receive Transtinib, gefitinib and the vehicles. Reagents were diluted with 0.5% lactic acid and were given by oral gavages. Tumor size was measured twice a week from an approximate mean starting volume of 0.32-0.42 cm^3^. The average tumor size was calculated by the formula π / 6 × (large diameter, mm) × (small diameter, mm)^2^. Statistical significance was evaluated using a one-tailed Student's t test.

### Experimental methods

#### General methods for the synthesis of the compounds

**7-fluoroquinazolin-4(3H)-one (2)**[25] A solution of 20 g of chemical **(1)** in 60 ml methanamide was heated to 130-140°C for 20 h, and then cooled to room temperature. The solid was collected and washed with ethanol and petroleum benzin then air-dried. MS (*m/z*) 164.5; ^1^H NMR (400 MHz, DMSO-*d6*) δ 12.37 (s, 1H), 8.16 (m, 2H), 7.37-7.73 (m, 2H) ppm.

**7-fluoro-6-nitroquinazolin-4(3H)-one (3)** A solution of 17 g of **(2)** in100ml H_2_SO_4_ was cooled to 0°C in an ice bath for over 30 min by intensely stirring and then a mixture solution of 20 ml of fuming nitric acid (fresh) and 20 ml of concentrated sulfuric acid was added dropwise. Then, the solution was reacted at room temperature with stirring for 1.5-2 h. The mixture was heated to 110°C with stirring for 2 h and poured onto ice with stirring. The solid was collected and washed with ice water and absolute ethyl alcohol, and air-dried. MS (*m/z*) 209.6; ^1^H NMR (400 MHz, DMSO-*d6*) δ 12.08-13.28 (b, 1H), 8.70-8.74 (d, 1H), 8.30 (s, 1H), 7.75-7.78 (d, 1H) ppm.

**7-ethoxy-6-nitroquinazolin-4(3H)-one (4)** 5.3 g of Na in a flask and 200 ml of anhydrous ethanol were added dropwise with reflux and stirring. After the Na was completely dissolved, stirring was continued for over 1 h, and 15.5 g of **(3)** was added. Heating at reflux was continued for 2.5 h. The reaction was cooled to room temperature, filtered and the solid was washed with ethanol and air-dried. MS (*m/z*) 233.6.

**4-chloro-7-ethoxy-6-nitroquinazoline(5)** [25] To 11.3 g of **(4)** in a flask, 88 ml of SOCl_2_ and 17 ml of POCl_3_ were added dropwise, respectively, and then the reaction was heated to over 80°C and intensely refluxed for 3.5 h. Then, the solution was cooled to room temperature, and the excess SOCl_2_ and POCl_3_ were removed by rotary evaporation. The solid residue was washed in toluene three times. The toluene was removed by rotary evaporation, and the solid was air-dried. MS (*m/z*) 254.8; ^1^HNMR (400 MHz, CDCl3-*d1*) δ 9.05 (s, 1H), 8.6 (s, 1H), 4.30-4.40 (d, 2H), 1.50-1.6 (m, 3H) ppm.

**N-(3-Y-4-X-phenyl)-7-ethoxy-6-nitroquinazolin-4-amine (6)a-c** A suspension of 13 g of **(5)** in 200 ml of isopropanol was stirred, and an equimolar of M was added. Next, the reaction was heated to over 85°C and refluxed for 4 h. After cooling to room temperature, the reaction was filtered and the solid was washed with isopropanol and air-dried. **6a** MS (*m/z*) 334.8, **6b** MS (*m/z*) 362.8, **6c** MS (*m/z*) 468.9.

**N-4--(3-Y-4-X-phenyl)-7-ethoxyquinazoline-4,6-diamine (7)a-c** A mixture of 13 g of **(6)**,200 ml of anhydrous ethanol, 35 ml of glacial acetic acid and 13 g of Fe power was stirred at 60°C for 1 h, and then the reaction was heated to reflux for 4 h. After cooling to room temperature, the reaction mixture was diluted with water and ethyl acetate with stirring for 15 min, and the resulting mixture was extracted with ethyl acetate. The combined organic layer was washed with saturated salt water. The excess glacial acetic acid was removed with saturated NaHCO_3_ 3 times, and the resulting organic was dried over anhydrous sodium sulfate and evaporated *in vacuo*. **7a** MS (*m/z*) 304.8, **7b** MS (*m/z*) 332.8, **7c** MS (*m/z*) 439.0.

**(S)-tert-butyl 2-formylpyrrolidine-1-carboxylate (9)** This compound was prepared from **(8)**, which is a commercially available chemical. A 1 L, 3-necked, round-bottomed flask equipped with an overhead stirred and a nitrogen inlet was charged with oxalyl chloride (14 ml) in dichloromethane (DCM, 200 ml). The solution was cooled to −78°C in a dry ice/ ethanol bath. A solution of DMSO (25 ml) in DCM was added dropwise. After 30 min, a solution of alcohol **(8)** (14 g) in DCM (100 ml) was added dropwise, and the reaction was continued for 1 h. Next, triethylamine (50 ml) was added dropwise and the mixture reacted for 30min. Then, the mixture was transferred to an ice/water bath and stirring was continued for 30 min. Then, the reaction was diluted with DCM and washed successively with water, 1 M HCl, saturated NaHCO3, and saturated NaCl. The DCM layer was dried over MgSO_4_, filtered and concentrated to afford **(9)** as an oil.

**(S)-tert-butyl 2-((E)-2-(ethoxycarbonyl)vinyl)pyrrolidine-1-carboxylate (10)** A 1 L, 3-necked, round-bottomed flask equipped with an overhead stirred and a nitrogen inlet was charged with NaH (10 g) in anhydrous tetrahydrofuran (THF, 100 ml). The solution was cooled in an ice/water bath for 20min, and then a solution of Triethyl phosphonoacetate (21.5 g) in THF (100 ml) was slowly added dropwise. A solution of crude **(9)** (16.4 g) in THF (100 ml) was added dropwise 1h later, and the reaction was continued for 1 h. After that, 100 ml of saturated NaCl was added dropwise to stop the reaction. The resulting solution was diluted with ethyl acetate and the layers were separated. The ether layer was washed with 1 M HCl and saturated NaCl, dried over anhydrous MgSO_4_, filtered and concentrated to afford **(10)** as a yellow oil that was used without further purification.^1^HNMR (CDCl_3_, 300 MHz) δ 6.92-6.76 (m, 1H),5.82 (d,1H), 4.56-4.32 (m, 1H), 4.25-4.12 (m, 2H), 3.48-3.27 (m, 2H), 2.20-1.98 (m, 1H), 1.91-1.72 (m, 2H), 1.43 (s, 9H), 1.25 (t,3H) ppm.

**(E)-3-((S)-1-(tert-butoxycarbonyl)pyrrolidin-2-yl)acrylic acid (11)** To a solution of **(10)** (26.4 g) in anhydrous ethanol (200 ml), LiOH (10 g) in water (125 ml) was added, and the solution was stirred at room temperature for 15 h. Ethanol was distilled off, and the residue dissolved in water (100 ml). The solution was cooled with an ice/water bath and carefully acidified with dilute HCl. The aqueous layer was extracted with DCM. The organic layer was dried over anhydrous MgSO_4_, filtered and concentrated to afford **(11)**. ^1^HNMR (CDCl_3_, 300 MHz) δ 6.71-6.91 (m, 1H), 5.81 (d, 1H), 4.26-4.38 (m, 1H), 4.16 (q, 2H), 3.40 (brm, 2H), 1.95-2.10 (m, 1H), 1.70-1.90 (m, 3H), 1.4 (s, 9H) and 1.25 (t, 3H) ppm.

**(S)-tert-butyl 2-((E)2-(4-(3-Y-4-X-phenylamino)-7-ethoxyquinazolin-6-ylcarbamoyl)vinyl) pyrrolidine -1-carboxylate (12)a-c** This compound was prepared from **(7)** (0.1 mol) and **(11)** (0.2 mol). To a solution of **(11)** in chloroform (60 ml) was added. After that one drop of N, N-dimethylformamide (DMF) was added as a catalytic agent. Then, the solution was stirred in an ice/water bath for over 20 min. A solution of oxalyl chloride (2.5 ml) in chloroform (40 ml) was added dropwise, and the reaction was stirred for 20 min and transferred to heat, 50°C for 2 h. To the flask, **(7)** was added, and the reaction was heated under reflux for 13 h. After cooling to room temperature, the suspension was filtered, and the liquid was washed with 5% of NaOH, 1 M HCl and water, dried over anhydrous MgSO_4_, filtered and concentrated to afford **(12)a-c. 12a MS (*m/z*) 526.1; 12b MS (*m/z*) 555.9; 12c MS (*m/z*) 662.1, 12a** ^1^H NMR (400 MHz DMSO-*d6*) δ9.72 (s, 1H), 9.58 (s, 1H), 8.88 (s, 1H), 8.53 (s, 1H), 8.01 (s, 1H), 7.87-7.89 (d, 1H), 7.37-7.38 (m, 1H), 7.26 (s, 1H), 7.21 (d, 1H), 6.72-6.78 (m, 1H), 6.39-6.43 (d, 1H), 4.11-4.41 (m,5H), 3.17 (s, 4H), 2.08 (s, 1H), 1.75-1.83 (m, 3H), 1.23-1.46 (m,9H) ppm.

**(E)-N-(4-(3-Y-4-X-phenylamino)-7-ethoxyquinazolin-6-yl)-3-((S)-pyrrolidin-2-yl)acrylamide (13)a-c** Compound **(12)** was dissolved in anhydrous dichloromethane (100 ml). The solution was stirred at room temperature, and 20-50% CF_3_COOH (50 ml) [26] was added. After 1 h of stirring, the reaction was evaporated to dryness in vacuo. The residue was taken up in ethyl acetate, washed with saturated NaHCO3, dried over anhydrous MgSO_4_, filtered and evaporated in vacuo.13a MS (m/z) 427.1; 13c, MS (m/z) 561.9; 13b MS (m/z) 456.1; 13b ^1^HNMR (400 MHz, DMSO-d6) δ 9.78 (s, 1H), 9.53 (s, 1H), 8.91 (s, 1H), 8.52 (s, 1H), 8.13-8.15 (m, 1H), 7.79-7.81 (m, 1H), 7.39-7.43 (m, 1H), 7.26 (s, 1H), 6.67-6.69 (m, 2H), 4.26-4.31 (m, 2H), 4.09-4.10 (m, 1H), 3.15-3.17 (m, 2H), 3.04-3.08 (m, 1H), 2.82-2.87 (m, 1H), 2.77-2.79 (m, 1H), 1.74-1.76 (m, 1H), 1.47 (m, 3H), 1.14 (s, 1H).

## CONCLUSIONS

In summary, we present an original, structurally distinct, irreversible EGFR-TKI, **13a-c**, that offers a number of advantages over earlier EGFR TKI agents, including high cellular potency and superior efficacy in relevant disease models. In particular, Transtinib, irreversibly and selectively targets both sensitizing and resistant-L858R/T790M mutant EGFR with activity toward the interactive residues (Met790/793 and Cys797) of the nucleotide binding pocket, showing promising therapeutic responses in both tumor cells and mouse models. In the H1975 xenograft model, oral dosing of Transtinib at 5 mg/kg daily for 4 weeks led to significant tumor growth inhibition without observed loss in body weight. Our docking results also suggested that Transtinib could bind well in the ATP binding pocket of the EGFR tyrosine kinase in the C-helix out inactive mode. This promising data for Transtinib strongly supports its selection as a clinical candidate.

## References

[1] Pao W, Chmielecki J (2010). Rational, biologically based treatment of EGFR-mutant non-small-cell lung cancer. Nat Rev Cancer.

[2] Maemondo M, Inoue A, Kobayashi K, Sugawara S, Oizumi S, Isobe H, Gemma A, Harada M, Yoshizawa H, Kinoshita I, Fujita Y, Okinaga S, Hirano H (2010). Gefitinib or chemotherapy for non–small-cell lung cancer with mutated EGFR. N Engl J Med.

[3] Lynch TJ, Bell DW, Sordella R, Gurubhagavatula S, Okimoto RA, Brannigan BW, Harris PL, Haserlat SM, Supko JG, Haluska FG, Louis DN, Christiani DC, Settleman J (2004). Activating mutations in the epidermal growth factor receptor underlying responsiveness of non–small-cell lung cancer to gefitinib. N Engl J Med.

[4] Paez JG, Jänne PA, Lee JC, Tracy S, Greulich H, Gabriel S, Herman P, Kaye FJ, Lindeman N, Boggon TJ, Naoki K, Sasaki H, Fujii Y (2004). EGFR mutations in lung cancer: correlation with clinical response to gefitinib therapy. Science.

[5] Mitsudomi T, Morita S, Yatabe Y, Negoro S, Okamoto I, Tsurutani J, Seto T, Satouchi M, Tada H, Hirashima T, Asami K, Katakami N, Takada M (2010). Gefitinib versus cisplatin plus docetaxel in patients with non-small-cell lung cancer harbouring mutations of the epidermal growth factor receptor (WJTOG3405): an open label, randomised phase 3 trial. Lancet Oncol.

[6] Mok TS, Wu YL, Thongprasert S, Yang CH, Chu DT, Saijo N, Sunpaweravong P, Han B, Margono B, Ichinose Y, Nishiwaki Y, Ohe Y, Yang JJ (2009). Gefitinib or carboplatin–paclitaxel in pulmonary adenocarcinoma. N Engl J Med.

[7] Rosell R, Carcereny E, Gervais R, Vergnenegre A, Massuti B, Felip E, Palmero R, Garcia-Gomez R, Pallares C, Sanchez JM, Porta R, Cobo M, Garrido P (2012). Erlotinib versus standard chemotherapy as first-line treatment for European patients with advanced EGFR mutation-positive non-small-cell lung cancer (EURTAC): a multicentre, open-label, randomised phase 3 trial. Lancet Oncol.

[8] Zhou C, Wu YL, Chen G, Feng J, Liu XQ, Wang C, Zhang S, Wang J, Zhou S, Ren S, Lu S, Zhang L, Hu C (2011). Erlotinib versus chemotherapy as first-line treatment for patients with advanced EGFR mutation-positive non-small-cell lung cancer (OPTIMAL, CTONG-0802): a multicentre, open-label, randomised, phase 3 study. Lancet Oncol.

[9] Kobayashi S, Boggon TJ, Dayaram T, Janne PA, Kocher O, Meyerson M, Johnson BE, Eck MJ, Tenen DG, Halmos B (2005). EGFR mutation and resistance of non–small-cell lung cancer to gefitinib. N Engl J Med.

[10] Yun CH, Boggon TJ, Li Y, Woo MS, Greulich H, Meyerson M, Eck MJ (2007). Structures of lung cancer-derived EGFR mutants and inhibitor complexes: mechanism of activation and insights into differential inhibitor sensitivity. Cancer cell.

[11] Stamos J, Sliwkowski MX, Eigenbrot C (2002). Structure of the epidermal growth factor receptor kinase domain alone and in complex with a 4-anilinoquinazoline inhibitor. J Biol Chem.

[12] Wood ER, Truesdale AT, McDonald OB, Yuan D, Hassell A, Dickerson SH, Ellis B, Pennisi C, Horne E, Lackey K, Alligood KJ, Rusnak DW, Gilmer TM (2004). A unique structure for epidermal growth factor receptor bound to GW572016 (Lapatinib) relationships among protein conformation, inhibitor off-rate, and receptor activity in tumor cells. Cancer Res.

[13] Tan F, Shen X, Wang D, Xie G, Zhang X, Ding L, Hu Y, He W, Wang Y, Wang Y (2012). Icotinib (BPI-2009H), a novel EGFR tyrosine kinase inhibitor, displays potent efficacy in preclinical studies. Lung Cancer.

[14] Kwak EL, Sordella R, Bell DW, Godin-Heymann N, Okimoto RA, Brannigan BW, Harris PL, Driscoll DR, Fidias P, Lynch TJ, Rabindran SK, McGinnis JP, Wissner A (2005). Irreversible inhibitors of the EGF receptor may circumvent acquired resistance to gefitinib. Proc Natl Acad Sci USA.

[15] Yun CH, Mengwasser KE, Toms AV, Woo MS, Greulich H, Wong KK, Meyerson M, Eck MJ (2008). The T790M mutation in EGFR kinase causes drug resistance by increasing the affinity for ATP. Proc Natl Acad Sci USA.

[16] Li D, Ambrogio L, Shimamura T, Kubo S, Takahashi M, Chirieac LR, Padera RF, Shapiro GI, Baum A, Himmelsbach F, Rettig WJ, Meyerson M, Solca F (2008). BIBW2992, an irreversible EGFR/HER2 inhibitor highly effective in preclinical lung cancer models. Oncogene.

[17] Solca F, Dahl G, Zoephel A, Bader G, Sanderson M, Klein C, Kraemer O, Himmelsbach F, Haaksma E, Adolf GR (2012). Target binding properties and cellular activity of afatinib (BIBW 2992), an irreversible ErbB family blocker. J Pharmacol Exp Ther.

[18] Kalous O, Conklin D, Desai AJ, O'Brien NA, Ginther C, Anderson L, Cohen DJ, Britten CD, Taylor I, Christensen JG, Slamon DJ, Finn RS (2012). Dacomitinib (PF-00299804), an irreversible Pan-HER inhibitor, inhibits proliferation of HER2-amplified breast cancer cell lines resistant to trastuzumab and lapatinib. Mol Cancer Ther.

[19] Ramalingam SS, Blackhall F, Krzakowski M, Barrios CH, Park K, Bover I, Seog Heo D, Rosell R, Talbot DC, Frank R, Letrent SP, Ruiz-Garcia A, Taylor I (2012). Randomized phase II study of dacomitinib (PF-00299804), an irreversible pan–human epidermal growth factor receptor inhibitor, versus erlotinib in patients with advanced non–small-cell lung cancer. J Clin Oncol.

[20] Wood ER, Shewchuk LM, Ellis B, Brignola P, Brashear RL, Caferro TR, Dickerson SH, Dickson HD, Donaldson KH, Gaul M, Griffin RJ, Hassell AM, Keith B (2008). 6-Ethynylthieno [3, 2-d]-and 6-ethynylthieno [2, 3-d] pyrimidin-4-anilines as tunable covalent modifiers of ErbB kinases. Proc Natl Acad Sci USA.

[21] Pettit GR, Srirangam JK, Barkoczy J, Williams MD, Boyd MR, Hamel E, Pettit RK, Hogan F, Bai R, Chapuis JC, McAllister SC, Schmidt JM (1998). Antineoplastic agents 365. Dolastatin 10 SAR probes. Anticancer Drug Des.

[22] Patel AB, Malpass JR (2008). Potential nicotinic acetylcholine receptor ligands from 2, 4-methanoproline derivatives. J Med Chem.

[23] George R, Srirangam JK cancer inhibitory peptides WO96/14856 1996-05-23.

[24] Morris GM, Huey R, Lindstrom W, Sanner MF, Belew RK, Goodsell DS, Olson AJ (2009). AutoDock4 and AutoDockTools4: Automated docking with selective receptor flexibility. J Comput Chem.

[25] Alexandre FR, Berecibar A, Wrigglesworth R, Besson T (2003). Novel series of 8H-quinazolino [4, 3-b] quinazolin-8-ones via two Niementowski condensations. Tetrahedron.

[26] Gururaja TL, Ramasubbu N, Levine MJ (1996). Solid-phase synthesis of human salivary mucin-derived O-linked glycopeptides. Letters in peptide science.

[27] Rabindran SK, Discafani CM, Rosfjord EC, Baxter M, Floyd MB, Golas J, Hallett WA, Johnson BD, Nilakantan R, Overbeek E, Reich MF, Shen R, Shi XQ (2004). Antitumor activity of HKI-272, an orally active, irreversible inhibitor of the HER-2 tyrosine kinase. Cancer Res.

[28] Gendreau SB, Ventura R, Keast P, Laird AD, Yakes FM, Zhang WT, Bentzien F, Cancilla B, Lutman J, Chu F, Jackman L, Shi YC, Yu PW (2007). Inhibition of the T790M gatekeeper mutant of the epidermal growth factor receptor by EXEL-7647. Clinical Cancer Res.

[29] Cross DA, Ashton SE, Ghiorghiu S, Eberlein C, Nebhan CA, Spitzler PJ, Orme JP, Finlay MRV, Ward RA, Mellor MJ, Hughes G, Rahi A, Jacobs VN (2014). AZD9291, an irreversible EGFR TKI, overcomes T790M-mediated resistance to EGFR inhibitors in lung cancer. Cancer Discov.

